# Developments in the Tools and Methodologies of Synthetic Biology

**DOI:** 10.3389/fbioe.2014.00060

**Published:** 2014-11-26

**Authors:** Richard Kelwick, James T. MacDonald, Alexander J. Webb, Paul Freemont

**Affiliations:** ^1^Centre for Synthetic Biology and Innovation, Imperial College London, London, UK; ^2^Department of Medicine, Imperial College London, London, UK

**Keywords:** synthetic biology, engineering biology, design cycle, tools, standardization

## Abstract

Synthetic biology is principally concerned with the rational design and engineering of biologically based parts, devices, or systems. However, biological systems are generally complex and unpredictable, and are therefore, intrinsically difficult to engineer. In order to address these fundamental challenges, synthetic biology is aiming to unify a “body of knowledge” from several foundational scientific fields, within the context of a set of engineering principles. This shift in perspective is enabling synthetic biologists to address complexity, such that robust biological systems can be designed, assembled, and tested as part of a biological design cycle. The design cycle takes a forward-design approach in which a biological system is specified, modeled, analyzed, assembled, and its functionality tested. At each stage of the design cycle, an expanding repertoire of tools is being developed. In this review, we highlight several of these tools in terms of their applications and benefits to the synthetic biology community.

## Introduction

The synthetic biology toolkit has expanded greatly in recent years, which can be attributed to the efforts of a highly dynamic community of researchers, ambitious undergraduate students in the International Genetically Engineered Machine competition (iGEM), and the growing number of amateur scientists from the DIY BIO movement. Each of these groups has bold ambitions for the rapidly growing field of synthetic biology, which aims to rationally engineer biological systems for useful purposes (Purnick and Weiss, 2009; Anderson et al., 2012; Landrain et al., 2013; Jefferson et al., 2014). The merging of several foundational sciences, including molecular, cellular, and microbiology with a set of engineering principles, is a profound shift and is the key distinction between synthetic biology and genetic engineering (Andrianantoandro et al., 2006; Heinemann and Panke, 2006; Khalil and Collins, 2010; Kitney and Freemont, 2012). Indeed, many social scientists, who are themselves a part of the synthetic biology community, have extensively explored the ontological implications of this perspective (Schark, 2012; Preston, 2013). Although many of the social aspects of synthetic biology are beyond the scope of this review, they will continue to shape the synthetic biology toolkit. In particular, society is an important stakeholder that has some influence over chassis (host cell) choice, the design of biosafety measures, biosecurity considerations, and long-term research applications (Marris and Rose, 2010; Anderson et al., 2012; Agapakis, 2013; Moe-Behrens et al., 2013; Wright et al., 2013; Douglas and Stemerding, 2014).

From a biological perspective, there have been important developments in the field across several areas, some of which have been reviewed elsewhere (Arpino et al., 2013; Lienert et al., 2014; Way et al., 2014). For instance, the number, quality, and availability of biological parts (bioparts, e.g., promoters and ribosomal binding sites) have continued to increase. This is exemplified by the iGEM student registry of standard biological parts, which has increased its biopart collection to include over 12,000 parts, across 20 different categories (partsregistry.org). However, due to its open nature, the iGEM registry contains parts of variable quality that are mostly uncharacterized. There are also professional parts registries, such as those at BIOFAB, which include expansive libraries of characterized DNA-based regulatory elements (Mutalik et al., 2013a,b). Although libraries of bioparts are indeed useful, putting them together into predictable devices, pathways and systems are incredibly challenging as many biological design rules are not yet fully understood (Endy, 2005; Kitney and Freemont, 2012). Developing synthetic passive and active insulator sequences may help increase predictability and thus reduce context dependency (Davis et al., 2011; Lou et al., 2012; Qi et al., 2012; Mutalik et al., 2013a). Notwithstanding these challenges, the field is progressing across several areas. One such area is biopart characterization, which is critical to the field, primarily because it is fundamentally a realization of several of the core engineering principles adopted in synthetic biology, namely standardization, modularization, and abstraction. Discrete biological parts of known sequence and behavior can be abstracted based upon a descriptive function and thus, their true complexity can be masked behind a biological concept. For example, discrete DNA sequences (bioparts) that fit a standardized descriptive function, such as a promoter, can be functionally characterized and as a consequence bioparts become reusable (modularization) for use in other synthetic systems. Additionally, methods that provide standardized ways of assembling DNA parts such as the BioBrick standard can help establish platforms for the sharing and reuse of bioparts. At a higher level, abstraction and standardization are important because they permit the separation of design from assembly (Endy, 2005).

A desirable consequence of this perspective is that these engineering principles enable the separation of labor, expertise, and complexity at each level of the design hierarchy (Endy, 2005). In practical terms, this separation of biological design from DNA assembly enables innovation within these hierarchies to occur at different rates. For instance, it is generally true that with more recent DNA assembly methods it is currently easier to assemble multi-part genetic circuits consisting of several bioparts, or even entire genomes, than it is to reliably predict how these bioparts will interact in the final system (Purnick and Weiss, 2009; Ellis et al., 2011; Arpino et al., 2013; Ellefson et al., 2014). However, it is envisioned that this will change, with the increasing adoption of high-throughput characterization platforms that can test entire biopart libraries in parallel. These platforms typically use automated liquid-handling robots, coupled with plate readers although microfluidics approaches are also gaining traction (Lin and Levchenko, 2012; Boehm et al., 2013; Benedetto et al., 2014). In either case, when coupled with automated data analysis, modeling, and sophisticated forward-design strategies (Marchisio and Stelling, 2009; Wang et al., 2009; Esvelt et al., 2011; Ellefson et al., 2014; Marchisio, 2014; Stanton et al., 2014), these high-throughput platforms provide the basis for the rapid prototyping workflows required to realize a synthetic biology design cycle (Kitney and Freemont, 2012).

**Figure 1:**
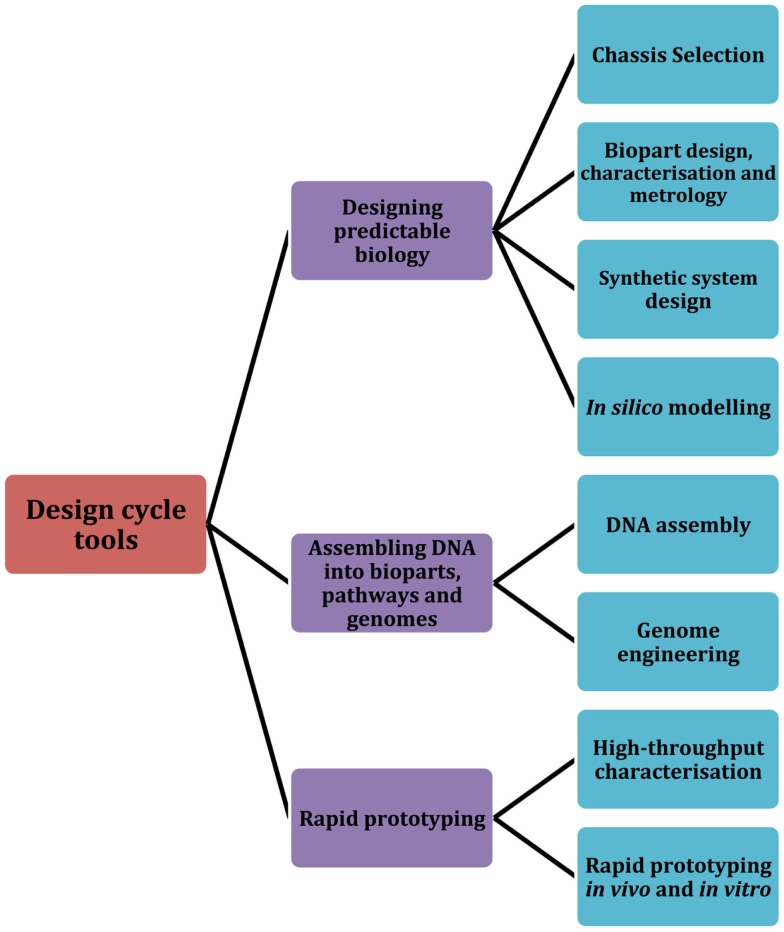
**Synthetic Biology Index of Tools and Software (SynBITS)**. A schematic summary of the synthetic biology design cycle tools as depicted in SynBITS (www.synbits.co.uk), an online community-managed index of synthetic biology tools and software.

In this review, we focus on several significant tools, both classical and emerging, that the field of synthetic biology employs as part of a typical design cycle workflow. Building upon a design cycle template, the review is organized to explore prominent tools and research methodologies across three core areas: designing predictable biology (design), assembling DNA into bioparts, pathways, and genomes (build), and rapid prototyping (test) (Figure 1). We first describe several of the core challenges that are associated with designing predictable biology, including the complexities associated with chassis selection, biopart design, engineering, and characterization. In parallel, we highlight relevant tools and methodologies that are particularly aligned with the engineering principles of synthetic biology. We then discuss established and newly developed DNA assembly methodologies, and group them according to four broad assembly strategies: restriction enzyme-based, overlap-directed, recombination-based, and DNA synthesis. Finally, we highlight several emerging rapid prototyping technologies that are set to significantly improve the field’s capacity for testing synthetic parts, devices, and systems. We conclude with a summary of several of the core challenges that were described in each of the design, build, test sections of the review and discuss whether the synthetic biology toolbox is equipped to address them. In addition to this, we have also created an online community, the Synthetic Biology Index of Tools and Software (SynBITS) – http://www.synbits.co.uk, which has also been structured according to the design cycle (Figure 1).

## Designing Predictable Biology

From an engineering perspective, living systems can be perceived as overly complex, inefficient, and unpredictable (Csete and Doyle, 2002). It is this perception that has driven the concept of the biopart, in which a particular DNA sequence is defined by the function that it encodes (Endy, 2005). Thus, complex biological functions can be conceptually separated (abstracted) from the complexities of the sequence context from which they originated (Endy, 2005). As a consequence of this approach, biological pathways and circuits can potentially be redesigned into less complex and potentially more predictable designs. The defining examples of this perspective are the toggle switch (Gardner et al., 2000), a genetic circuit defined by two repressible promoters that were engineered to form a mutually inhibitory network, and the repressilator (Elowitz and Leibler, 2000), a type of oscillator (biological clock). What sets these examples apart from general genetic engineering is that modeling was used to predict and optimize the behavior of these genetic circuit designs prior to their construction.

While these forward-design approaches were hugely successful, the repressilator displayed noisy behavior as a result of stochastic fluctuations in components of the genetic circuit (Elowitz and Leibler, 2000). In other words, *in silico* modeling did not fully capture the true *in vivo* complexity of the synthetic circuit. Likewise, the toggle switch experienced natural fluctuations in gene expression that were sufficient to create variations in the level of inducer needed to switch the cells from one state to another. These variations were also not fully anticipated during *in silico* modeling (Gardner et al., 2000). While these genetic circuits have been improved, with novel oscillator (Stricker et al., 2008; Olson et al., 2014) and toggle switch designs, including those designed for mammalian cells (Muller et al., 2014b) and plants (Muller et al., 2014a), it is clear that the modeling of biological systems still requires a concerted and long-term effort. Critical to this effort is the availability of new synthetically designed bioparts and experimental data that accurately captures the behavior of the components or bioparts that constitute a synthetic system (Arkin, 2013) as well as the characteristics or influence that the chassis/host cell enacts upon them.

### Chassis selection

As an engineering concept, the chassis refers to a physical internal framework or structure that supports the addition of other components that combine to form a finalized engineered structure. From a synthetic biology perspective, the concept invokes an understanding that a biological chassis is a tool to provide the structures that accommodate (host) the execution of a synthetic system, including the provision of a metabolic environment, energy sources, transcription, and translation machinery, as well as other minimal cellular functions (Acevedo-Rocha et al., 2013; Danchin and Sekowska, 2014). Chassis selection is therefore a critical design decision that synthetic biologists are required to take, particularly since the chassis will directly influence the behavior and function of a synthetic system. Essentially, the chassis determines which bioparts can be used since they must be compatible with the biological machinery that is present. This can result in a difficult choice for the synthetic biologist: either to use an established chassis and design the circuit to be orthogonal with that host, or design a synthetic system that fits a requirement and then choose a host chassis that is compatible with the resultant bioparts or system. These constraints can to some degree be designed around, either by engineering the chassis to knockout genes that optimize its orthogonality and reduce burden, through codon optimization (Chung and Lee, 2012) or through the use of insulator sequences that negate context dependency effects (Guye et al., 2013; Torella et al., 2014a). Ultimately, however, chassis selection will dictate the downstream design considerations for any given synthetic system, and therefore, chassis selection must be coordinated with biopart design efforts.

In order to rationalize which chassis selection strategy is most appropriate for an intended application, it is important to consider the consequences and advantages of each strategy. Where a chassis is selected as a priority above that of the design considerations of the synthetic system, it is important to consider whether the chassis has been extensively characterized in the literature and/or if the chassis has known intrinsic capabilities that complement the intended application (Table 1). Additionally, access to detailed biological knowledge of a chassis will aid modeling-guided design efforts and the implementation of chassis optimization strategies for dealing with burden or metabolic flux effects. Likewise, the wealth of knowledge acquired about model organisms across several biological disciplines may encourage synthetic biologists to consider them as a potential chassis in preference to established favorites (Table 1). Indeed, there are already several emerging chassis that are gaining traction and are set to be utilized more frequently in the field (Table 1).

**Table 1:** **Synthetic biology chassis**.

Chassis	Advantages	Disadvantages
**ESTABLISHED CHASSIS**		
*Bacillus subtilis*	Model Gram-positive organism. Generally regarded as safe (GRAS) organism. Genetically tractable and genome sequences are available. Secretion of proteins. Extensive range of molecular biology tools are available, e.g., plasmids (Harwood et al., 2013; Radeck et al., 2013). Rapid growth, inexpensive to grow and maintain, can be induced to form heat and desiccation resistant spores (Harwood et al., 2013). Spores can be transported easily and cheaply. Suicide mechanisms are available (Wright et al., 2013).	Non-integrative plasmids are not always stably maintained between cell generations. Protease-deficient strains are required to minimize proteolytic degradation of expressed proteins.
Cell-free protein synthesis (CFPS)/transcription–translation coupled reactions (TX–TL)	Protein/metabolite production is decoupled from the need of the cell to survive and reproduce – ideal if product is toxic or inhibitory to living chassis. Amenable to high-throughput workflows (Sun et al., 2013a,b).	The biological system does not self-reproduce
		Reactions typically only last 4–6 h due to depletion of the reaction energy mix and/or the accumulation of inorganic phosphates. Reaction components can also be expensive
		Variability between cell extract batches
*Escherichia coli*	Genetically tractable and genome sequences are available. An extensive range of molecular biology tools are available, e.g., plasmids, phages, etc. Rapid growth, inexpensive to grow and maintain, extensive range of published data relating to this chassis, suicide mechanisms available (Wright et al., 2014). Whole-cell metabolic models have been developed and are being improved (Atlas et al., 2008).	Few post-translational modifications compared to eukaryotes, e.g., reduced protein glycosylation
*Saccharomyces cerevisiae*	Glycosylation of expressed proteins. Genetically tractable and genome sequenced. Molecular biology tools are available, e.g., plasmids	The core oligosaccharides that comprise the protein glycosylation events in *S. cerevisiae* are thought to be responsible for the hyper-antigenic nature of proteins expressed in this chassis rendering them potentially unsuitable for therapeutic uses (Hamilton and Gerngross, 2007; Cregg et al., 2009; Walsh, 2010).
**EMERGING CHASSIS**		
*Chlamydomonas reinhardtii*	An established model organism; eukaryotic photosynthetic organism	Slow cultivation time. Several strains have a cell wall, and are therefore difficult to transform. Low transformation frequency due to genome integration of plasmids
*Geobacillus sp*.	Several strains currently being developed as synthetic biology chassis including *G. thermoglucosidasius* (Bartosiak-Jentys et al., 2013). Enables the application of metabolic and enzymatic processes at higher temperatures (e.g., 55–65°C optimum for *G. thermoglucosidasius*) than is possible with alternative chassis	Few biological parts have been characterized. The majority of antimicrobial drugs are unstable at the high temperatures that these chassis can grow at, thus limited cloning strategies are available.
Induced pluripotent stem cells (iPSCs)	An ethical source of stem cells for therapeutic and other responsible innovation applications (Cachat and Davies, 2011; Ye et al., 2013). Potential platform for engineering complex synthetic systems across multi-tissue structures	Cellular differentiation programs are not yet fully understood and therefore rational engineering is difficult
*Marchantia polymorpha*	Compared with other plant model organisms, this chassis has a relatively simple, “streamlined” genomic architecture. Genome projects are underway and several molecular biology tools are in development. Can be cultured easily and grows rapidly (Sharma et al., 2014)	Molecular biology tools are still in development (Chiyoda et al., 2014).
*Physcomitrella patens*	An established model organism for research on plant evolution, development, and physiology (Schaefer and Zryd, 1997; Nishiyama, 2000). Genome sequence available. Does not have a codon usage bias	Slow growth; month timescale. Low transformation efficiency
*Pichia pastoris*	Higher heterologous protein expression and reduced glycosylation compared to *S. cerevisiae*. Successful expression of more than 200 heterologous proteins has been published. Proteins expressed in this chassis are thought to be less antigenic in nature than those produced in *S. cerevisiae* making this organism more suitable for therapeutic protein generation. *P. pastoris* is a methylotroph, and can therefore, grow with methanol as a sole carbon source. Can grow to high cell densities with high growth rates on inexpensive media (Cereghino, 2000; Cregg et al., 2000; Vogl et al., 2013).	High level of clonal variation (Aw and Polizzi, 2013). Few plasmid vectors are available
*Protocells*	Enables a complete bottom-up approach in which the cellular machinery, metabolism, genome, etc., can all be bespoke engineered (Chen et al., 2004; Xu et al., 2010)	Still under development
*Synechocystis sp*.	*Cyanobacteria sp*. are model organisms for the study of photosynthesis, as well as carbon and nitrogen fixation (Heidorn et al., 2011). Synthetic biology approaches may enable coupling of photosynthesis with the generation of biofuels and natural products (Depaoli et al., 2014). Tools are available for use in this chassis (Heidorn et al., 2011; Berla et al., 2013).	Specialized growth conditions are required (Berla et al., 2013)
Synthetic yeast 2.0	First designer eukaryotic genome but based on an established chassis – *S. cerevisiae*. One of the first bespoke-engineered chassis, with a defined genetic context, that has been rationally engineered for the benefit of the synthetic biology community (Annaluru et al., 2014; Lin et al., 2014). As part of the project, LoxPsym recombination sites are being added to the 3′ end of all non-essential genes to allow inducible genome shuffling using a system called SCRaMbLE (Dymond and Boeke, 2012)	The alteration of the natural genome structure may negatively affect genome stability
		Complex biosafety, biosecurity, and ethical challenges may arise as a consequence of alterations in the natural functions of *S. cerevisiae*
		Project not yet complete
**POTENTIAL CHASSIS**		
*Caenorhabditis elegans*	Genetically tractable (Redemann et al., 2011) and the genome have been sequenced. The number and position of every cell during development are known and therefore this organism has great potential for the engineering of whole-organism, developmentally organized synthetic systems. Used in synthetic screens (O’Reilly et al., 2014)	Genetic lines have to be maintained
*Danio rerio*	Regenerative abilities. The organism is largely transparent and therefore expression of fluorescent reporter systems can be used to characterize *in vivo* synthetic systems. Established systems biology model (Mushtaq et al., 2013)	There may be alternative chasses that are more appropriate for some applications due to the ethical and legal considerations associated with the use of vertebrates in research
*Drosophila melanogaster*	Genetically tractable, genome sequenced, and proven relevance to human disease models. *Drosophila*-derived cell lines can be engineered for constitutive and inducible expression of proteins (Yang and Reth, 2012)	Genetic lines have to be maintained
*Xenopus tropicalis*	Genome sequenced. Used in synthetic screens (White et al., 2011; Tomlinson et al., 2012).	There may be alternative chasses that are more appropriate for some applications due to the ethical and legal considerations associated with the use of vertebrates in research

Alternatively, a synthetic system could be specified and designed as a priority above that of chassis selection. As a consequence, there will be chassis, which are not compatible with the synthetic system and others that may require extensive engineering to accommodate its design. However, this approach is complementary to those chassis that are bespoke engineered. “Synthia,” the first organism to feature a fully synthetically manufactured genome, is indicative that the field of synthetic biology is shifting toward the development of rationally engineered chassis (Gibson et al., 2010a). Though it is important to recognize that the “Synthia” genome, while synthetic in origin, was not designed to significantly alter the characteristics of the chassis, and therefore, does not represent the first truly bespoke-engineered chassis. Yet, its successors, the synthetic yeast project (Annaluru et al., 2014), protocell developments (Xu et al., 2010), and even to some extent cell-free expression systems (Shin and Noireaux, 2012; Sun et al., 2013a) may all usher in an era in which the design of bespoke-engineered chassis is routine. Wholly rationally engineered chassis could conceivably be built around the specifications of a synthetic system, such that the chassis is both compatible with the synthetic system and the majority of its cellular resources are directed toward the execution of the synthetic system. In this sense, the function of the synthetic system would be free of chassis constraints; however, the full realization of this approach is still several decades away. Until then, chassis selection will remain a trade-off between which should be prioritized for each application, the chassis or the synthetic system? There are of course many other considerations to address, some of which we cover in the biopart design section of this review and others that have been previously discussed in the literature (Heinemann and Panke, 2006; Arpino et al., 2013; Danchin and Sekowska, 2014).

### Biopart design and engineering

The field of synthetic biology continues to benefit from decades of biological research that has built a knowledge base of biological systems that can be deconstructed and re-engineered as bioparts and synthetic systems. Here, we highlight prominent bioparts that are particularly aligned with the engineering principles of synthetic biology. In most cases, existing natural biological parts can be reused in synthetic devices or systems. However, there are situations where new bioparts need to be designed and synthesized by modifying existing bioparts or by creating entirely new parts *de novo*. These novel parts could be enzymes that catalyze unnatural reactions (Jiang et al., 2008; Rothlisberger et al., 2008), molecular biosensors (Penchovsky and Breaker, 2005), protein scaffold (Koga et al., 2012; Heider et al., 2014), DNA or RNA scaffolds (Rothemund, 2006; Delebecque et al., 2011), ribosome-binding sites with specifically designed transcription rates (Salis et al., 2009), promoters with novel regulatory features and/or specific translation rates (Marples et al., 2000; Kelly et al., 2009).

Transcriptional circuits use RNA polymerase operations per second (PoPS) as the common signal carrier but, until recently only a small set of DNA-binding proteins and associated operator sequences were used to regulate the flux of RNA polymerase (RNAP) and construct synthetic circuits. The lack of a large set of orthogonal regulatory proteins has limited the complexity of synthetic systems (Purnick and Weiss, 2009), but a new wave of engineered proteins has greatly increased the number of tools available to synthetic biology circuit designers. The clustered, regularly interspaced, short palindromic repeats (CRISPR)/Cas system consists of CRISPR and CRISPR associated genes (cas) coding for related proteins, which together constitute an adaptive prokaryotic immune system (Barrangou et al., 2007). The CRISPR loci consist of repeats interspaced with spacer sequences, which are transcribed and processed into crRNAs containing individual spacer sequences that are complementary to foreign DNA. The crRNAs bind Cas9 nuclease and the resulting complex recognizes and cleaves sequences complementary to the spacer sequences. This natural system has been repurposed as a transcriptional regulator by modifying the Cas proteins to deactivate nuclease activity and creating artificial guide RNA (gRNA) sequences to create the CRISPR interference (CRISPRi) system. Deactivated Cas9:gRNA complexes act as repressors by binding specific sites and inhibiting RNAP activity (Qi et al., 2013). Alternatively, Cas9 can be fused to domains that recruit RNAP in order to act as transcriptional activators (Bikard et al., 2013; Mali et al., 2013).

Transcription activator-like effectors (TALEs) are proteins secreted by *Xanthomonas* bacteria in order to activate expression of plant genes during the course of infection. They consist of tandem repeats of a small domain with two variable amino acid sites. The amino acid identities of the variable sites have a simple mapping to the DNA base recognized, enabling chains of domains to be stringed together in order to bind specific sequences (Boch et al., 2009; Moscou and Bogdanove, 2009). The simple modular nature of TALEs has enabled the engineering of synthetic proteins such as TAL effector nucleases (TALENs) (Mahfouz et al., 2011) and artificial orthogonal activators and repressors (Morbitzer et al., 2010; Blount et al., 2012).

Translation initiation regulators are relatively easy to *de novo* design as they rely on the reasonably well-characterized thermodynamics of RNA structure (Liang et al., 2011). However, unlike transcriptional circuits, there is no common signal carrier and thus they cannot be as easily composed into complex regulatory designs. By repurposing a regulatory element from the *tnaCAB* operon of *E. coli*, Liu et al. (2012), have created an adapter to convert translational regulators into transcriptional regulators (Liu et al., 2012). The 5′-end of the operon codes for a short leader peptide, TnaC that stalls the ribosome in the presence of free tryptophan. The stalled ribosome then blocks a Rho factor-binding site located adjacent to the stop codon of *tnaC*, allowing the transcription of the downstream genes *tnaA* and *tnaB*. The ribosome-binding site of *tnaC* in the native operon is constitutive but replacing this with translational regulator sequences, such as the RNA-IN/OUT system (Ross et al., 2013), enables the control of transcription of downstream genes.

In recent years, there has been rapid progress in developing software algorithms to enable the design of synthetic proteins that can be controlled at the atomic level of resolution (Leaver-Fay et al., 2011). Computational protein design is generally split into two components. Initially, a backbone scaffold is either artificially generated or taken from an existing known structure. Secondly, the amino acid sequence is optimized such that it minimizes the free energy of folding. It appears that minimizing a potential energy function by trialing different amino acid identities and rotamers is sufficient to achieve this. There have been a number of dramatic successes using this approach including the *de novo* design of enzymes. The design of a novel enzyme requires knowledge about the transition state structure of the reaction to be catalyzed and a predicted spatial arrangement of chemical groups that are likely to stabilize the transition state. The transition state structure and the stabilizing constellation of chemical groups around it can be designed theoretically (theozyme) (Tantillo et al., 1998), and using this knowledge, known protein structures can be searched for sites capable of accommodating side chain functional groups in the desired geometry (Zanghellini et al., 2006). These methods have resulted in a number of successful enzyme designs (Jiang et al., 2008; Rothlisberger et al., 2008).

In the cell, many biochemical processes are spatially organized in order to locally concentrate substrates or isolate toxic substances (e.g., the carboxysome or peroxisome) and reduce cross talk between components. Efforts to engineer high-level organization in synthetic biological systems is a major challenge with applications in encapsulating artificial organelles or protocells (Choi and Montemagno, 2005; Agapakis et al., 2012; Hammer and Kamat, 2012; Mali et al., 2013), the precise detection and delivery of payloads (Sukhorukov et al., 2005; Uchida et al., 2007). Methods based on the computational protein design methods described above have been applied to create new self-assembling biomaterials at the atomic level from protein subunits that do not naturally form into higher-order structures (King et al., 2012, 2014). Other work has focused on using hydrophobic patterning of peptides to produce higher-order structures based on coiled-coils (Rajagopal and Schneider, 2004; Woolfson and Mahmoud, 2010; Zaccai et al., 2011; Fletcher et al., 2013). However, these are not designed to atomic level accuracy, tend to be chemically synthesized and so have not yet been reported to assemble *in vivo*. An alternative approach is to reuse naturally occurring protein–protein interfaces and assemblies (Padilla et al., 2001; Howorka, 2011; Sinclair et al., 2011), although ultimately it may be more desirable to design completely artificial protein scaffolds that are more likely to be biologically neutral and avoid the Mullerian complexity of naturally evolved biological systems (Dutton and Moser, 2011).

Novel protein biomaterials have applications in metabolic engineering by co-locating enzymes in the same pathway on a structural scaffold. This has the advantage of increasing the local concentration of substrates improving reaction kinetics, helping to prevent the loss of intermediates to competing pathways and the accumulation of toxic intermediates (Dueber et al., 2009). Protein cages can be used to completely encapsulate metabolic pathways and create synthetic bacterial micro-compartments. In a recent example, genes from the propanediol utilization operon (*pdu*) encoding for an empty protein shell in *Salmonella enterica* were expressed in *E. coli*. Short peptide sequences known to bind to *pdu* shell proteins were used to target pyruvate decarboxylase and alcohol dehydrogenase to the micro compartment resulting in increased ethanol production (Lawrence et al., 2014). This approach promises to be particularly useful for biosynthetic pathways involving toxic metabolites.

Similarly, structural scaffolds can be constructed using nucleic acids. Base pairing in nucleic acids makes predicting and designing structures somewhat more tractable than for proteins. For example, 2D and 3D structures have been engineered *in vitro* using long single stranded DNA (ssDNA) and small ssDNA oligonucleotides called “staples,” that direct the folding of the long ssDNA into a pre-designed structure (Rothemund, 2006; Douglas et al., 2009; Han et al., 2011). It has also been shown to be possible to express simpler nanostructures *in vivo* using RNA transcribed by the cell (Delebecque et al., 2011). These structures were used together with specific protein-binding aptamers to efficiently channel substrates from one enzyme to another and substantially increase hydrogen production. At short distances, substrate channeling has been found to be more effective than expected by simple 3D Brownian diffusion models (Fu et al., 2012, 2014).

A number of tools for predicting and designing relative translation rates of ribosome-binding sites have been developed (Reeve et al., 2014) including the RBS Calculator (Salis et al., 2009; Salis, 2011), the RBS Designer (Na and Lee, 2010), and the UTR Designer (Seo et al., 2013), which can aid operon design (Arpino et al., 2013). These software tools are based on thermodynamic models of the pre-initiation complex of the 30S ribosomal subunit and the messenger RNA (mRNA) and include terms based on the free energy required to unfold the unbound mRNA, the free energy of hybridization of the mRNA and the 16S rRNA, and various other terms. If the pool of free 30S ribosomal subunits is assumed to be roughly constant then the translation initiation rate can be assumed to be proportional to exp(−βΔG). Mechanistic predictive models for promoters are somewhat more complicated as promoter strength is related to the binding of the sigma factor and RNAP, and also the efficiency of promoter escape. However, there has been some success in predicting the strength of promoters for the *E. coli* sigma factor σ^E^ using relatively simple position weight matrix models (Rhodius and Mutalik, 2010; Rhodius et al., 2012).

Most of the synthetic regulatory tools described above are used in the construction of transcriptional circuits. Nevertheless, post-transcriptional circuit design, particularly using RNA molecules, has attracted a great deal of interest in recent years (Liang et al., 2011; Wittmann and Suess, 2012). Unlike proteins, RNA molecules are somewhat easier to design due to their well-understood thermodynamics and the dominance of secondary structure formation on folding. One important application area is the use of RNA as switches (riboswitches) that respond to their environment. Riboswitches are RNA molecules that can regulate protein production in response to changes in the concentration of a small molecule and occur naturally as well as being synthetically designed. These molecules are composed of an RNA aptamer that binds a specific small-molecule ligand. On binding the small molecule, the RNA aptamer may then change conformation resulting in either the occlusion of the Shine–Dalgarno sequence or its increased accessibility. Expression of the downstream genes is then turned either on or off in response (Suess et al., 2004). Alternatively, aptamers may be coupled to a ribozyme that allosterically cleaves itself in response to ligand binding (Tang and Breaker, 1997; Penchovsky and Breaker, 2005). The *de novo* design of small-molecule binding RNA aptamers is a non-trivial task but novel aptamers can be evolved *in vitro* using methods such as SELEX (systematic evolution of ligands by exponential enrichment) (Ellington and Szostak, 1990; Tuerk and Gold, 1990; Jenison et al., 1994). Riboswitches may have uses in metabolic engineering such as down-regulating upstream genes if a metabolite reaches toxic levels (Zhang and Keasling, 2011). RNA aptamers have also found use as mimics of GFP by binding small-molecule fluorophores (Paige et al., 2011). As discussed in the metrology section, these aptamers can be used to monitor mRNA levels.

### Biopart characterization

Biopart characterization describes the functional and experimental metadata that is required to sufficiently capture the biological behavior of a biopart and the context in which it is being tested. The type and range of these characterization data have evolved over time as highlighted by refinements in biopart characterization data sheets (Arkin, 2008; Canton et al., 2008). Typically, these experimental metadata include information on the plasmid vector, the testing organism or strain, any relevant growth conditions, and the equipment or methodologies used to capture the bioparts functionality. The primary purpose of biopart characterization data is to provide the necessary experimental data for predictive *in silico* biological modeling. The determination of which biological data provide0 the greatest insight into the behavior of a given biological system is largely debatable; at least until more biological design rules are understood. The context dependency of bioparts *in vivo* provides significant challenges in predicting their function as modular components. Therefore, for biopart characterization, measurement standards should largely be defined by those biological data that can be measured (metrology), how relevant those data are for predicting the behavior of a biological process (modeling) and how widely these data can be adopted (standardization). This last point is particularly important since bioparts should ideally be reusable (modular) across multiple applications and contexts. To enable this, the formatting of these data should ideally be standardized to facilitate the measurement and use of biopart characterization data across different *in silico* design tools, forward-design strategies, and workflows.

The most concerted effort is the Synthetic Biology Open Language (SBOL) consortia, a group of life scientists, engineers, computer scientists and mathematicians that are actively building a set of standards that define a common data format for bioparts and their accompanying characterization data (Bower et al., 2010; Galdzicki et al., 2011; Quinn et al., 2013; Roehner and Myers, 2013). The concept is to create a file structure that can capture biopart sequence, characterization and experimental data in a format that is platform independent. Crucially, the format is designed to be extendable to include additional parameters as new characterization technologies and methodologies emerge. In combination with SBOL visual (SBOLv), which defines a standardized way to visually denote bioparts through symbols, the SBOL standard is set to enable the seamless sharing of genetic designs. Several bioinformatics and molecular cloning design tools have already adopted SBOL, and the intention for SBOL is to provide an interoperable standard between several *in silico* tools such that individuals can optimize their workflow as required, yet retain information between them. Several of these *in silico* tools have been extensively reviewed (MacDonald et al., 2011; Galdzicki et al., 2014); however, we include an updated list here, that combines *in silico* tools from these existing reviews, along with several new tools, in particular R2o designer and COOL (Table 2).

**Table 2:** **Emerging tools for the forward-design of synthetic pathways and systems**.

Software tool	Description
**PATHWAY AND CIRCUIT DESIGN**	
AutoBioCAD	Automated design of gene regulatory circuits (Rodrigo and Jaramillo, 2013).
Cell designer	Modeling of biochemical networks. http://www.celldesigner.org/
Genetic engineering of cells (GEC)	Biological programing language and visual simulator of biological systems. http://research.microsoft.com/en-us/projects/gec/
GenoCAD	GenoCAD is an open-source computer-assisted-design (CAD) application for synthetic biology. http://www.genocad.org/
Genome compiler – iGEM edition	Cloud based genetic design tool that is optimized for BioBrick assembly and the iGEM competition. http://igem.genomecompiler.com/join
MATLAB: Simbiology	SimBiology^®^ provides an application and programmatic tools to model, simulate, and analyze dynamic biological systems. http://www.mathworks.co.uk/products/simbiology/
Operon calculator	Rational design of bacterial operons to control protein expression. https://salis.psu.edu/software/OperonCalculator_EvaluateMode
OptCom	A modeling framework for the flux balance analysis of microbial communities. http://maranas.che.psu.edu/software.htm
ProMoT	Process Modeling Tool, software for the construction and manipulation of complex technical and biological systems. http://www.mpi-magdeburg.mpg.de/projects/promot/
**BIOPART DESIGN**	
CaDNAno	Simplifies the process of designing three-dimensional DNA origami nanostructures. http://caDNAno.org/
COOL	Codon Optimization OnLine (COOL): a web-based multi-objective optimization platform for synthetic gene design (Chin et al., 2014)
mfold/UNAfold	Prediction of nucleic acid secondary structure (Markham and Zuker, 2008). http://mfold.rna.albany.edu/
NUPAC	Prediction and design of nucleic acid secondary structure (Zadeh et al., 2011). http://www.nupack.org/
Promoter calculator	*E. coli* σ^E^ – In development (Rhodius and Mutalik, 2010; Rhodius et al., 2012).
RBS calculator	The Ribosome-Binding Site (RBS) Calculator is a design method for predicting and controlling translation initiation and protein expression in bacteria. https://salis.psu.edu/software
RBS designer	Computational design of synthetic ribosome-binding sites (RBS) to control gene expression levels. http://ssbio.cau.ac.kr/web/?page_id=195
RNA designer	Designs RNA secondary structure (Andronescu et al., 2004). http://www.rnasoft.ca/cgi-bin/RNAsoft/RNAdesigner/rnadesign.pl
Rosetta	Tools for structure prediction, design, and remodeling of proteins and nucleic acids. http://maranas.che.psu.edu/software.htm
UTR designer	Predictive design of mRNA translation initiation region to control prokaryotic translation efficiency (Seo et al., 2013). http://sbi.postech.ac.kr/utr_designer
**MISCELLANEOUS**	
R2oDNA designer	Designs orthogonal biologically neutral linker sequences for DNA assembly and other uses (Casini et al., 2013, 2014). http://r2oDNA.com/
SBOL	SBOL core provides an interoperable data format to transfer biopart characterization data between software programs and tools (Roehner and Myers, 2013). http://www.sbolstandard.org/
SBOLv	SBOL visual defines a standardized way to visually denote bioparts through symbols (Quinn et al., 2013). http://www.sbolstandard.org/visual

In contrast to SBOL, which is still under development, the iGEM registry of standard biological parts (http://parts.igem.org/) has provided a relatively large-scale and publically accessible repository of bioparts and some biopart characterization data for almost 10 years. Since its inception, the iGEM community has led, with mixed success, concerted efforts to improve the quality of its characterization data. The 2014 iGEM competition, for instance, has announced several specialist awards for teams that demonstrate advancements in metrology. This push for improvements in biopart characterization at the grassroots (undergraduate) level has permeated up to professional characterization efforts. For instance, early difficulties in the reproducibility of the behavior of DNA regulatory elements between iGEM teams and professional research groups provided the context for the emergence of the relative promoter unit (RPU) as a reference measurement standard (Kelly et al., 2009). The RPU standard compares the relative activity of a promoter against a reference standard, tested under the same experimental conditions, with an RPU arbitrarily set to 1. The rationale underpinning this standard is that while the absolute activity of a promoter may differ between experimental repeats, the relative activity should be less prone to such variability. Essentially, a promoter that is twice the strength of the standard should remain so, even between different experimental conditions and methodologies of different research groups. In agreement with this, Kelly et al. (2009) reported a 50% decrease in variability, when RPUs were independently reported for a set of Anderson constitutive promoters. Inter-experimental variability and reproducibility of data are a significant problem facing all scientific endeavors (Collins and Tabak, 2014), and for synthetic biologists the RPU measurement standard has highlighted these issues within the context of biopart characterization.

There are, however, no universally agreed standards for advancing biopart characterization metrology, though in general the field is shifting away from relative measurements toward absolute measurements (Table 3). Many research groups are currently interested in measuring absolute numbers of cells, DNA molecules, proteins, or other components that constitute the synthetic system and its context. But this shift is largely incremental as certain types of biological data are very difficult to measure directly. These challenges are, however, worth addressing since it is assumed that such biological data are essential to improve the predictive capabilities of forward-design *in silico* models (Bower et al., 2010; Cooling et al., 2010). Yet, because of such data limitations, current-modeling approaches often depend upon inferred or assumed parameters that are derived from biological data that can be experimentally verified. One such modeling approach by Canton et al. (2008), proposed a set of standardized measurement units termed, PoPs and ribosomes per second (RIPS), even though the absolute biological data that underpin them has not been directly measured *in vivo* (Canton et al., 2008; Cooling et al., 2010; Marchisio, 2014). PoPs infers the flow of RNAP along a point of DNA per second and RIPS infers the flow of ribosomes across an mRNA molecule. As previously noted, PoPs and RIPS cannot be measured directly; instead they are calculated using fluorescence data from a reporter protein (e.g., GFP), growth data (OD), and largely assumed values for other parameters including protein or mRNA concentrations. These data are generally measured *in vivo* within a plate reader setup, though flow cytometry-based characterization efforts are increasingly being adopted and are set to progress metrology at the single cell level (Díaz et al., 2010; Tracy et al., 2010; Choi et al., 2013; Zuleta et al., 2014). In either case, if experimental setups are sufficiently standardized, it is possible to convert measurements between several widely adopted standards: RPU, PoPs/RIPS, and absolute measurements such as GFP cell^−1^ s^−1^ (Kelly et al., 2009).

**Table 3:** **Synthetic biology measurement standards**.

Measurement standard	Advantages	Disadvantages
**RELATIVE**		
Relative promoter unit (RPU)	Reduces variability between promoter characterization data across different laboratory groups, equipment or slightly different experimental protocols.	The choice of reference standard promoter requires consensus
	Concept may be applied in other contexts beyond promoter characterization.	
**ABSTRACT**		
Polymerase operations per second (PoPs) and ribosomes per second (RIPS)	Describes information flow (input/output) from transcriptional-based logic devices	Units cannot be directly measured
		May not capture biological processes at the mechanistic level
	Abstract level modeling	Does not describe biological information that is sent through other mechanisms e.g., protein post-translational modifications
**ABSOLUTE**		
GFP cell^−1^s^−1^	Direct measurement of the number of fluorescent reporter proteins produced	Requires careful consideration of the design and measurement of the calibration curve needed to compare fluorescence (arbitrary units) and known fluorescent protein concentrations
	Direct comparisons can be made between data sets	
	Concept may be applied to other biological reporters	

Notwithstanding the above limitations of PoPs and RIPS, these units were primarily designed to reflect the behavior of genetic circuits at the level of information flow (inputs/outputs) rather than at the truly mechanistic level (Gardner et al., 2000; Canton et al., 2008; Stricker et al., 2008; Marchisio, 2014). For biologists, however, these terms represent an abstract merger of several elements of the transcriptional and translational machinery, which does not accurately reflect the mechanistic underpinning biology. However, abstract and mechanistic modeling approaches are not necessarily mutually exclusive since both approaches can provide insightful information for the forward-design of predictable biological pathways and systems.

Advances in metrology and novel measurement standards that are accessible, and hence, more widely adopted will clearly benefit the whole field of synthetic biology. Yet, it is challenging to achieve consensus for developing measurement standards, since standards intrinsically empower those that promote them above those that have not adopted them (Calvert, 2012; Frow and Calvert, 2013). Conversely, it should be noted that consensus in measurement standards and metrology does not preclude innovation if such standards are flexible enough to accommodate developments in the tools and methodologies that enable researchers to easily share, reuse, and build upon existing genetic designs. Likewise, standardized biological information can still be combined with expert knowledge, or novel forward-design strategies for the construction of complex, robust, and efficient biological systems.

Metrology in biology has been enabled in part to continual advancements in microscopy and in synthetic biology, technologies such as microfluidics coupled with quantitative microscopy are continuing to gain traction (Lin and Levchenko, 2012; Song et al., 2013; Walter and Bustamante, 2014). Microfluidic technologies enable the precise manipulation of fluids at small-scales through engineered channels, chambers, and valves. Microfluidic chip designs are sufficiently advanced to enable a high-degree of spatial-temporal control of liquid-flows to and between individual cells or cell populations seeded within the chambers of prefabricated microfluidics chips. With this level of control, small molecules that induce gene expression or influence other biological processes can be precisely delivered to elicit acute, basal, or morphogenic responses. Within a synthetic biology context, such systems have been used to characterize DNA regulatory elements, intercellular communication, and synthetic pathways at high spatial–temporal resolution. One notable example shown by Hansen and O’Shea (2013), in which the microfluidic control of the delivery of a small molecule (1-NM-PP1) was used to control the nuclear localization of a Yeast stress-inducible transcription factor, Msn2. Deliberate alterations in the oscillatory or acute dynamics of Msn2 trans-nuclear localization revealed the extent to which promoters respond differently to transcriptional-activation dynamics. From this, promoters could be modeled *in silico*, according to the extent that they could elicit differential gene expression patterns, as a consequence of their ability to distinguish a genuine nuclear-influx of Msn2 from background “noise” (Hansen and O’Shea, 2013). Manipulation of these dynamics could be used to reduce promoter leakiness; or conversely to exploit different classes of promoter transcriptional-signal processing to coordinate multiple genetic programs, through the modulation of a single transcription factor.

Another important technology for synthetic biology is flow cytometry, which relies upon hydrodynamic focusing to guide single cells through a fluidic channel where they are measured (Piyasena and Graves, 2014). Recent models of flow cytometers can simultaneously measure cell size, complexity, and up to 17 channels of fluorescence (Basiji et al., 2007; Piyasena and Graves, 2014), each of which could be used to capture data from different reporter outputs. Of the biological reporters available, RNA aptamers are particularly noteworthy, since they have the potential to increase the type and range of biological information that can be measured (Cho et al., 2013; Pothoulakis et al., 2014). For instance, several groups have reported the simultaneous measurement of both transcription (mRNA levels) and translation (protein levels) (Chizzolini et al., 2013; Pothoulakis et al., 2014). In both cases Spinach, an RNA aptamer that binds a fluorophore (Paige et al., 2011), was incorporated within the 3′ untranslated region (UTR) of a fluorescent reporter protein, either GFP or RFP (Chizzolini et al., 2013; Pothoulakis et al., 2014). Providing there is no spectral-overlap between fluorophores, this strategy could conceivably be up-scaled to measure entire synthetic pathways, and thus inform operon design strategies (Hiroe et al., 2012; Chizzolini et al., 2013). Metabolic engineering efforts may also benefit from engineered RNA aptamer-hybrids that simultaneously bind cellular metabolites and a fluorophore, effectively enabling the real-time reporting of intracellular metabolic flux (Barrick and Breaker, 2007; Roth and Breaker, 2009; Sefah et al., 2013; Szeto et al., 2014). These exponential increases in biological data could significantly impact whole-cell modeling (Atlas et al., 2008; Gama-Castro et al., 2011; Shuler et al., 2012; O’Brien et al., 2013) and pave the way for novel measurement standards or modeling approaches that are wholly based upon directly measured biological processes.

## Assembling DNA into Bioparts, Pathways, and Genomes

Recombinant DNA technology, in which DNA sequences are “cut and pasted” together via restriction enzymes and DNA ligases respectively, form the foundations of the 1970s biotechnological revolution and have greatly expanded the possibilities of genetic engineering (Zimmerman et al., 1967; Cohen et al., 1973; Lobban and Kaiser, 1973). Synthetic biology continues to benefit from these foundational advancements in recombinant DNA-based biotechnology. For example, the BioBrick DNA assembly standard, uses a set of standardized restriction sites, termed the prefix (*Eco*RI *Xba*I) and suffix (*Spe*I *Pst*I), that flank each biopart (BioBrick) (Rokke et al., 2014). Digestion and ligation using these sites allow several parts to be assembled together in a standard fashion. The BioBrick standard was originally developed by Tom Knight in 2003 and is still used within the synthetic biology community, particularly during the iGEM competition (Rokke et al., 2014). The BioBrick assembly standard is beneficial to the synthetic biology community for several reasons. Firstly, the flanking restriction site sequences set a physical border that defines individual bioparts. As a result, the BioBrick assembly standard realizes the idea that DNA sequences encode discrete functions and that these individual blocks (BioBricks) can be assembled together like “lego^tm^ bricks.” Additionally, the use of standardized restriction sites ensures that the cloning strategy for assembling BioBricks is standardized across the entire research community; thereby eliminating the requirement for some cloning-based tacit knowledge. Despite these advantages, a major limitation of the approach is that BioBrick sequences must not contain the prefix and suffix restriction sites, thus limiting the range of sequences that can be assembled. Additionally, when *Xba*I and *Spe*I sites are ligated together, the ligated sequence creates a “scar,” which does not contain either an *Xba*I or *Spe*I restriction site (Speer and Richard, 2011; Rokke et al., 2014). Scar sequences may alter the behavior of the flanking bioparts or prevent the generation of fusion proteins, and therefore, can be undesirable (Anderson et al., 2010; Ellis et al., 2011).

**Figure 2:**
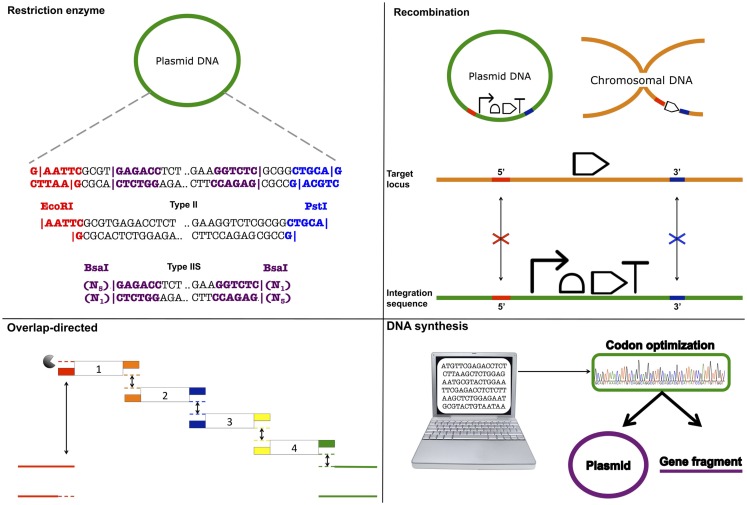
**DNA assembly strategies**. Restriction enzyme – restriction enzymes recognize specific DNA sequences and either cut within their recognition sequence (Type II) or adjacent to its recognition sequence (Type IIS) to create sticky or blunt-ended DNA fragments that can be ligated to other DNA fragments. Recombination – cellular DNA repair and recombination machinery can be utilized to integrate a DNA construct within a specific genomic locus. Integration is guided through 5′ and 3′ sequence complementarity of the integration sequence with the target locus. Overlap-directed – assembly order is guided by 20–40 bp overlaps at the ends of each DNA fragment that share sequence homology with adjacent DNA fragments. In the case of Gibson assembly, these homologous ends are processed (chew-back) and fused together (anneal) via the sequential activity of an exonuclease, a ligase, and a polymerase. DNA synthesis – DNA sequences are designed and optimized *in silico* for *de novo* synthesis. Commercial constructs are delivered as gene fragments or are pre-cloned within a plasmid vector.

BioBrick assembly is also an inefficient way to create large multi-part constructs since it is limited to the assembly of two bioparts per reaction, as defined by the three antibiotic (3A) assembly method (Speer and Richard, 2011). ePathBrick potentially overcomes this limitation through the use of an expansive set of BioBrick-compatible isocaudomer pairs of restriction sites (Xu et al., 2012). The combinatorial assembly of multiple inserts is possible through the restriction digestion and ligation of different isocaudomer pairs into an ePathBrick vector. Backwards compatibility with the BioBrick standard is certainly advantageous from the perspective of modularity (re-useable bioparts); however, ePathBrick is still subject to the BioBrick limitations of forbidden sequences and post-assembly scar sequences. With these limitations in mind, several DNA assembly methods have been developed to address them (Figure 2) (Chao et al., 2014).

### Restriction-directed assembly: Bgl bricks, golden gate, and SEVA

Golden gate assembly (Engler et al., 2008; Engler and Marillonnet, 2011, 2013), Bgl Bricks (Anderson et al., 2010), and the Standard European Vector Architecture (SEVA) (Silva-Rocha et al., 2013) use a set of restriction sites to standardize DNA assembly. However, in contrast to the BioBrick standard, these assembly methods use rare restriction site sequences, and therefore, support a greater range of sequences. The Bgl Brick standard uses *Bgl*II and *Bam*HI restriction sites. Annealed *Bgl*II and *Bam*HI restriction sites generate an inert, glycine-serine encoding scar sequence, which in contrast to the BioBrick standard scar allows the assembly of protein fusions. Golden Gate assembly supports scar-less assembly through the use of Type IIS restriction enzymes that act by cleaving outside of their recognition sequence leaving a variable overhang, which directs the assembly order and ligation reaction. If cleavage sites are designed appropriately, these overhangs can be designed so that the final assembled sequences are “scar-less.” More recently, combinatorial Golden Gate assembly methods have been described that allow multi-gene constructs, including synthetic pathways, to be assembled in parallel (Engler and Marillonnet, 2011, 2013). SEVA and to some extent ePathBrick, differ from the majority of assembly methods in that they are more correctly described as modular standards. SEVA describes a set of criteria for the physical assembly of plasmids according to a three-component architecture: an origin of replication segment, a selection marker segment, and a cargo segment (Silva-Rocha et al., 2013). These segments are flanked by insulator sequences and assembled together with a set of rare restriction sites. While the rationales for restriction site-based assembly methods support modularity, their limitations have led several research groups in the synthetic biology community to “trade-in” standardization and modularity, in favor of “bespoke” assembly methods that enable one-pot assembly of multiple DNA parts.

### Overlap-directed assembly: Gibson, SLiC, CPEC, SLiCE, and PaperClip

Daniel Gibson developed a widely adopted DNA assembly method that allows multiple DNA fragments to be assembled in a one-pot *in vitro* reaction (Gibson et al., 2009; Gibson, 2011). The Gibson assembly uses a linearized destination vector and PCR generated inserts as its starting material. Inserts are generated with PCR primers that include 20–40 bp overlaps that share sequence homology to adjacent DNA fragments. As a result, the correct arrangement of several inserts entering the same destination vector can be defined. During the reaction, a T5 exonuclease acts to chew-back at the 5′ ends of the linearized destination vector and inserts. The reaction occurs at 50°C and therefore the T5 exonuclease along with its activity is eventually inactivated. The destination vector and inserts anneal together, as defined by their exonuclease exposed homologous ends, and Phusion polymerase activity acts to fill in the gaps. Finally, Taq ligase seals nicks between the joined DNA fragments. Gibson assembly is simple, can assemble five or more parts in a single reaction, and the reaction itself only takes around 60 min, after which the final assembled product can be directly transformed into *E. coli*.

Sequence and Ligase-independent Cloning (SLiC) (Li and Elledge, 2007), Circular Polymerase Extension Cloning (CPEC) (Quan and Tian, 2009, 2011), Seamless Ligation Cloning Extract (SLiCE) (Zhang et al., 2012) are also overlap-directed DNA assembly methods that all result in the same final product. Therefore, inserts and destination vectors designed for Gibson assembly can also be used in SLiC, CPEC, and SLiCE assemblies. During SLiC reactions, the destination vector and inserts are independently treated *in vitro* with T4 DNA polymerase, which exhibits exonuclease activity in the absence of deoxynucleotide triphosphates (dNTPs). Exonuclease activity is subsequently inhibited with the addition of deoxycytidine triphosphate (dCTP) and the destination vector and inserts are then mixed together for annealing. However, because SLiC reactions do not include DNA ligase, gaps, or nicks in the DNA are repaired once the final product is transformed into *E. coli*. CPEC on the other hand, is a PCR-based approach in which the linearized destination vector and inserts are initially denatured to produce single DNA strands. These are then annealed together, as directed by the homologous DNA overlap regions. Once annealed, the destination vector and inserts act to prime each other for extension via the activity of Phusion DNA polymerase. A low number of PCR cycles act to prevent the propagation of PCR-based errors. SLiCE reactions markedly differ from the assembly methods just described in that they involve an *ex vivo* bacterial cell extract (PPY, *E. coli* DH10B λ–red) as the reaction mix. Since exogenous polymerases and DNA ligases are not required, this is a potentially cost-effective method and like Gibson, assembly reactions also typically take just 60 min, although at 37°C instead of 50°C as per Gibson assembly.

PaperClip DNA assembly is a relatively new overlap-directed assembly method that uses pairs of bridging oligonucleotides termed “Clips” to direct the assembly of multi-part constructs (Trubitsyna et al., 2014). Interestingly, PaperClip assembly protocols are derived from CPEC (PCR-based) and SLiCE (*ex vivo*-based) assembly methodologies. Yet, PaperClip assembly is advantageous over these assembly methods in that once the “Clips” have been prepared, the required assembly order of parts can be determined in a single reaction. While, “Clips” introduce an alanine encoding scar sequence between each part, the bridging oligos used to assemble multi-part constructs in ligase cycling reaction (LCR) assembly are scar-less (Rouillard et al., 2004; de Kok et al., 2014). Though as we describe below, PaperClip assembly differentiates itself from Gibson, CPEC, SLiCE, and LCR assembly methods in that *de novo* assembly fragments do not need to be generated each time the order assembly is changed (Trubitsyna et al., 2014).

Overlap-directed assembly methods use sequence homology to guide assembly and are therefore largely sequence independent. This is a clear advantage over restriction site-based DNA assembly methods and their forbidden sequences. It should be noted that repeat and short DNA sequences, particularly those that give rise to DNA secondary structures, can reduce the efficiency of overlap-directed methods and are best avoided. On the other hand, CPEC denaturation PCR cycles mitigate the effect of DNA secondary structures to some degree. Overlap-directed methods are also efficient at assembling multiple parts in a predefined order within a single one-pot reaction. Gibson assembly, for example, has been used to assemble genome-scale DNA fragments, including the complete assembly of the *M. genitalium* genome (583 kb) and more recently the entire mouse mitochondrial genome (16.3 kb) (Gibson et al., 2008, 2010b). It is clear therefore that overlap-directed assembly methods can be scaled toward the assembly of large genetic constructs, including synthetic genomes (Gibson et al., 2010a). Yet, despite their proven utility, they are inherently “bespoke” and are thus in conflict with the ideals of embedding standardization and modularity concepts within DNA assembly strategies. For instance, custom primers are needed to generate inserts *de novo* each time the assembly order is changed and while it is now possible to automate overlap-directed assembly primer design (Hillson et al., 2012), these assembly methods still require tacit knowledge. To this end, additional methodologies are being developed with the aim of making overlap-directed DNA assembly modular.

### Overlap-directed assembly with biologically neutral linker sequences

Modular overlap-directed assembly with linkers (MODAL) makes use of standardized flanking sequences and biologically neutral (orthogonal) linkers as part of a modular overlap-directed DNA assembly strategy (Casini et al., 2013). MODAL assembly requires bioparts to be standardized with the addition of a common prefix and suffix sequence. The prefix and suffix sequences do not contain restriction sites and are not directly required for the assembly process. Instead, these sequences serve as a consistent set of PCR primer “landing pads” that enable all MODAL bioparts to be generated using the same primer set. Additionally, these sites serve as priming sites for the PCR-directed addition of biologically neutral linker sequences that serve as homologous sequences for overlap-directed assembly. These sequences can be designed with R2oDNA Designer (Casini et al., 2013, 2014), an *in silico* tool that was developed to automatically design orthogonal linker sequences for use in MODAL and other applications. Similar strategies have also been developed in parallel, in which biologically inactive unique nucleotide sequences (UNSes) were utilized to guide the Gibson assembly of insulated genetic circuits (Guye et al., 2013; Torella et al., 2014a,b). These neutral sequences are often standardized and may also incorporate BioBrick restriction sites, thus enabling modularity and standardization to be embedded within overlap-directed assembly strategies.

### *In vivo* DNA assembly and genome engineering

An array of chassis with a broad set of useful, extensively characterized genotypes and phenotypes are available to the synthetic biology community (Table 2). However, there are applications where it is appropriate to rationally engineer a chassis. For instance, an application may require a novel strain that is optimized, at the genome level, to fit a set of specific design requirements that may be difficult or otherwise impractical to bioprospect. Typically, genome-engineering efforts are geared toward maximizing compatibility between a chassis and a synthetic system, increasing the efficiency of the metabolic flux across a synthetic pathway or toward minimizing burden effects. The field is making progress in establishing rationally engineered genomes; of which the synthetic yeast 2.0 project (Dymond and Boeke, 2012; Annaluru et al., 2014; Lin et al., 2014) and minimal genome projects (Glass et al., 2006; Dewall and Cheng, 2011; Shuler et al., 2012), are currently the most prominent exemplars. These genome-engineering efforts are made possible due to the emergence and ongoing development of an expanding set of *in vivo* DNA assembly methods and genome-engineering tools.

Recombineering approaches, in which synthetic linear ds/ssDNA sequences are introduced into genomic regions through a process of homologous recombination, have proven utility as an efficient method to knockout or knock-in sequences of interest. Recombineering enables genomic engineering at all scales; from the introduction of single nucleotide polymorphisms, to the replacement of 40 kb+ DNA fragments or even toward the assembly of entire genomes (Narayanan and Chen, 2011; Zhao et al., 2011; Bonde et al., 2014; Song et al., 2014). *S. cerevisiae* transformation-associated recombination (TAR) cloning (Kouprina and Larionov, 2008), *Bacillus* Domino (Ohtani et al., 2012), and the *E. coli* Single-Selective-Marker Recombination Assembly System (SRAS) (Shi et al., 2013) uses the endogenous homologous recombination machinery of the indicated organisms to assemble DNA constructs *in vivo*. A variant of the yeast TAR method has successfully generated several genomes, including the first *in vivo* assembled synthetic genome of *M. genitalium* (Gibson et al., 2008). *Bacillus* domino has also shared similar successes in that this assembly method has also assembled DNA at the genomic scale, including the mouse mitochondrial genome and the rice chloroplast genome (Itaya et al., 2008; Ohtani et al., 2012; Iwata et al., 2013). While *E. coli* SRAS could potentially support the assembly of large DNA fragments, it is currently optimized for the assembly of multi-part constructs and their simultaneous integration into the *E. coli* genome (Shi et al., 2013).

The lambda-red (λ-red) recombinase system is another recombineering strategy, which is used for the integration of ssDNA or dsDNA constructs into the *E. coli* genome (Murphy, 1998; Murphy and Campellone, 2003). Optimized lambda-red recombination protocols can integrate linear DNA sequences into a specific genomic target, guided by only 35–50 bases of flanking homologous sequence (Murphy and Campellone, 2003). Interestingly, lambda-red-mediated recombination events do not require endogenous recombination proteins (e.g., RecA) and instead linear ssDNA or dsDNA constructs are integrated into the *E. coli* genome via the action of three λ-red proteins; Gam, Exo, and Beta. Gam protects linear dsDNA from the exonuclease activity of the endogenous proteins RecBCD, thus increasing the efficiency at which the introduced dsDNA will be recombined into the genome. λ-red-mediated recombination itself is primarily mediated by Exo, a 5′–3′ – dsDNA-specific exonuclease and Beta, a ssDNA annealing protein. It is interesting to note that Gam-associated protection of dsDNA is exploited in SLiCE *ex vivo* DNA assembly and as we discuss later, for *in vitro* transcription–translation (TX–TL) coupled reactions involving linear DNA as the input (Sitaraman et al., 2004).

The introduction of a large number of rationally engineered genomic changes is a potentially laborious process; however, multiplex automated genome engineering (MAGE) enables the automation of large-scale recombineering strategies. MAGE was originally characterized within EcNR2, a variant strain of *E. coli* MG1655. EcNR2 was modified to incorporate the λ-red recombination system and also to be deficient in DNA mismatch repair via the knockout of the *mutS* gene (Wang et al., 2009). MAGE relies upon the λ-red Beta protein-assisted incorporation of ssDNA oligonucleotides, typically 90mers, into the lagging strand during DNA replication (Wang et al., 2009). MAGE oligonucleotide pools can be designed to incorporate highly specific changes at a single genomic site, to introduce multiple changes across a single locus or to simultaneously target multiple genomic sites. These outcomes are largely defined through the diversity of the MAGE oligonucleotide pool, where mixtures of degenerate oligonucleotides can be designed to introduce divergent changes across a broad sequence and recombination efficiency space. Where a large number of simultaneous genomic changes are required, the process can be repeated through multiple MAGE cycles of cell growth, electroporation of oligonucleotides into the cell population, and phenotype/genotype characterization. MAGE cycles can be automated through a microfluidics-type setup and in combination with MODEST or optMAGE, which are *in silico* MAGE oligonucleotide design tools (Table 4), the directed evolution of a rationally designed chassis, can be accomplished within a timescale of several days. Indeed, MAGE has been used to optimize the DXP pathway in *E. coli*, such that isolated variants that are capable of a fivefold increase in lycopene production were engineered in just 3 days (Wang et al., 2009).

**Table 4:** **DNA assembly and genome-engineering tools**.

Assembly method	Mechanism	Sequence independent*	Scar-less assembly	Software support tools
Bgl Brick	Type II restriction enzymes	No	No	Under development
BioBrick standard	Type II restriction enzymes	No	No	Registry of standard biological parts, an online and physical repository of BioBrick parts (http://parts.igem.org/)
ePathBrick	Type II restriction enzymes	No	No	–
SEVA	Type II restriction enzymes	No	Possible	SEVA-DB platform, an online repository of SEVA-compliant parts (Silva-Rocha et al., 2013)
Golden gate	Type IIS restriction enzymes	No	Possible	j5, an automated primer design tool can be adapted for Golden gate combinatorial assembly (Hillson et al., 2012)
Gibson	Overlap-directed	Yes – however, short or repeat sequences that give rise to secondary DNA structures are a problem	Yes	j5, an automated primer design tool (Hillson et al., 2012)
SLiC	Overlap-directed	Yes – however, short or repeat sequences that give rise to secondary DNA structures are a problem	Yes	j5, an automated primer design tool (Hillson et al., 2012)
CPEC	PCR-based overlap-directed	Yes – however, short or repeat sequences are a problem	Yes	j5, an automated primer design tool (Hillson et al., 2012)
SLiCE	*Ex vivo* overlap-directed	Yes – however, short or repeat sequences that give rise to secondary DNA structures are a problem	Yes	j5, an automated primer design tool (Hillson et al., 2012)
PaperClip	Overlap-directed with oligonucleotide pairs “Clips”	Yes – however, constructs cannot contain repetitive parts or more than 40 bases of identical regions	No	–
Ligase cycling reaction	Bridging oligo-directed assembly	Yes	Yes	Gene2Oligo: oligonucleotide design for *in vitro* gene synthesis (Rouillard et al., 2004). http://berry.engin.umich.edu/gene2oligo/
Gibson with UNSes	Overlap-directed with orthogonal linkers	Yes	No	R2oDNA designer: computational design of biologically neutral (orthogonal) synthetic DNA sequences (Casini et al., 2013, 2014). Computational design rules for UNSes (Guye et al., 2013; Torella et al., 2014a,b).
MODAL	Overlap-directed with orthogonal linkers	No	No	R2oDNA designer: computational design of biologically neutral (orthogonal) synthetic DNA sequences (Casini et al., 2013, 2014).
*Bacillus* domino	*In vivo* homologous recombination	Yes – however, cannot assemble *Bacillus* genomic sequences	Yes	–
*E. coli* (SRAS)	*In vivo* homologous recombination	Yes – however, homologous sequences are needed for recombination	Yes	–
MAGE and CAGE	*In vivo* homologous recombination/conjugation	Yes – however, homologous sequences are needed for recombination	Yes	MAGE oligonucleotide design tools: MODEST (Colloms et al., 2014) http://modest.biosustain.dtu.dk/; optMAGE http://arep.med.harvard.edu/optMAGE/
Yeast TAR	*In vivo* homologous recombination	Yes	Yes	–
Engineered nucleases (zinc-finger nucleases, TALENs, and CRISPR/Cas9)	DNA cleavage and non-homologous end joining (NHEJ) or homology-directed repair (HDR)	Yes – cleavage can be directed toward sequence of interest	Yes*, However NHEJ can introduce random mutations.	E-CRISP: CRISPR target site identification (Heigwer et al., 2014). http://e-crisp-test.dkfz.de/E-CRISP/
SIRA	Serine integrase recombinational assembly	Yes – as long as ϕC31 recombination sites are avoided	No	Software support tools are in development (Colloms et al., 2014)
DNA synthesis	Polymerase cycling assembly from pools of overlapping custom oligos	Yes – however, repeat sequences or high GC content can be problematic	Yes	Codon optimization, the removal of undesirable restriction sites and the specification of 5′ and 3′ sequences are possible during the order processes of several commercial companies. GeneDesigner (Villalobos et al., 2006) https://www.DNA20.com/resources/genedesigner

*Sequence-independent assembly strategies do not place restrictions upon which DNA sequences are permitted within assembly fragments.

In parallel with MAGE, conjugative assembly genome engineering (CAGE) can be used to coordinate large-scale genomic engineering strategies across phases, such that subtle genetic combinations that are lethal can be screened out in a manner that does not impede overall progress toward the final strain. To achieve this, CAGE guides the conjugal transfer of MAGE genome modifications between hierarchical pairs of donor–recipient *E. coli*, such that a new strain emerges which incorporates all of the MAGE-optimized modifications from previous generations (Isaacs et al., 2011). Multiple MAGE–CAGE rounds enable a large set of genomic modifications to be generated and carefully integrated. As an example of such an approach, Isaacs et al. (2011) used a MAGE–CAGE strategy to replace 314 TAG stop codons with the synonymous TAA in *E. coli* across its entire genome (Isaacs et al., 2011).

Engineered nucleases, which cleave specific DNA sequences, creating double-stranded DNA breaks, can be used to introduce genomic changes. These strategies depend upon the random occurrence of perturbations in DNA repair mechanisms, where double-stranded breaks are inappropriately repaired, resulting in erroneous sequence insertions, deletions, or even significant chromosomal rearrangements. Screening strategies to identify cells that contain desirable genomic alterations can be subsequently isolated as an engineered population. Zinc-finger nucleases (Ellis et al., 2013), TALENS (Mahfouz et al., 2011), and the CRISPR/Cas system (Sander and Joung, 2014) have all been engineered for these types of genome editing applications. The CRISPR/Cas system is particularly interesting since as discussed above, a deactivated Cas9 nuclease:gRNA complex can also be fused with domains that act as transcriptional activators or repressors (Bikard et al., 2013; Mali et al., 2013; Qi et al., 2013). Nuclease-mediated genome editing strategies can also be combined with a recombineering-type approach, in which an engineered dsDNA can be introduced into the cell, which has sequence complementarity at the site of the nuclease breakage. Through the endogenous homologous recombination machinery (DNA repair mechanisms), it is possible to rationally integrate the engineered dsDNA into the genome (Cong et al., 2013; Sander and Joung, 2014). Thus in combination, MAGE, CAGE, and engineered, targeted nucleases (Zinc, TALENS and Cas9) represent a set of molecular tools that enable genome editing and the transcriptional control of natural and synthetic genomes.

### DNA synthesis

Synthetic biology has greatly benefited from the rapid decline in the cost of commercial gene synthesis, a phenomenon popularized by the Carlson curve (Carlson, 2009), which is analogous to Moore’s law. Although the rate of decline has decreased in recent years, with DNA synthesis costs now relatively stable (Carlson, 2009, http://www.synthesis.cc/cgi-bin/mt/mt-search.cgi?blog_id=1&tag=CarlsonCurves&limit=20), it is likely that new disruptive technologies will decrease DNA synthesis costs in the near future. DNA synthesis costs are still sufficiently low that many research groups routinely order the synthesis of genes and gene fragments although still prohibitive for library generation or for the synthesis of large multi-part pathways. In these cases, gene synthesis can be combined with additional cloning techniques such as overlap-directed assembly or mutagenic PCR to generate large constructs or biopart libraries, respectively. It is likely that as DNA synthesis costs decline, there will be a continual shift away from DNA assembly toward *de novo* DNA synthesis, which will have a transformative effect on synthetic biology and the design-build-test cycle.

## Rapid Prototyping

High-throughput platforms bring scalability to biopart characterization efforts, through the parallel characterization of function and context of entire biopart libraries (Arkin, 2013; Keren et al., 2013; Mutalik et al., 2013b). To ensure consistency at such scale, high-throughput workflows typically couple liquid-handling robots with plate readers (Keren et al., 2013), flow cytometry (Piyasena and Graves, 2014; Zuleta et al., 2014), or microfluidics (Lin and Levchenko, 2012; Benedetto et al., 2014) in order to automate the majority of the experimental workflow. Several high-throughput platforms have been described, the majority of which were used to characterize DNA regulatory elements (Keren et al., 2013; Mutalik et al., 2013a,b), however, this is expanding to include the characterization of enzymes (Choi et al., 2013), multi-gene operons (Chizzolini et al., 2013), and RNA aptamers (Cho et al., 2013; Szeto et al., 2014). When coupled with automated data analysis and modeling, these technologies and workflows could become rapid prototyping platforms, enabling a truly biological design cycle approach (Kitney and Freemont, 2012). At present, these high-throughput workflows are typically semi-rational design strategies in which thousands of biopart variants are tested and screened as part of a discovery workflow. Yet, at the same time, these approaches are simultaneously generating large data sets that provide useful insights into biological processes that may inform biological design rules. For example, characterization efforts have informed several systematic methodologies for the rational optimization of synthetic systems at the transcriptional, translational, and post-translational level (Table 2) (Arkin, 2008; Arpino et al., 2013; Reeve et al., 2014). In cases where synthetic systems could conceivably be rationally designed, it is still naïve to assume that the first iteration of a synthetic biological system will perfectly match the design specifications. Instead, multiple iterations of the design-build-test cycle will be needed until forward-design approaches are sufficiently advanced. Therefore, the requirements of interoperable standards in which researchers can apply the same protocols across different liquid-handling platforms are essential. To this end, Linshiz et al. (2013) have implemented a high-level robot programing language (PaR-PaR), which can translate biological protocols into instruction sets for an extendable range of liquid-handling robot platforms. As a consequence of this approach, the training requirements for end-users to implement the same biological protocol across different liquid-handlers are significantly reduced (Linshiz et al., 2013). If, as the authors propose, PaR–PaR is combined with SBOL, then the adoption of PaR–PaR scripts will enable researchers to share the same high-throughput DNA assembly or characterization protocols, but have them implemented across different experimental and equipment setups.

The majority of the rapid prototyping platforms that we have described so far have been optimized for testing biological parts, devices, and systems *in vivo*; however, *in vitro* systems are emerging as a useful testing platform. Cell-free protein synthesis (CFPS) systems based upon *E. coli* (Nirenberg and Matthaei, 1961; Sitaraman et al., 2004; Hong et al., 2014), *B. subtilis* (Zaghloul and Doi, 1987), *S. cerevisiae* (Hodgman and Jewett, 2013; Gan and Jewett, 2014), or other cell extracts have been reported in the scientific literature for several decades. Several CFPS systems are commercially available and are principally marketed as protein expression systems. Optimized *E. coli* CFPS systems can synthesize up to 2.3 mg/ml of the target protein (Caschera and Noireaux, 2014), including those that are toxic *in vivo*. In recent years, the synthetic biology community has repurposed CFPS systems as *in vitro* transcription–translation (TX–TL) coupled characterization platforms. A typical TX–TL reaction combines a synthetic system encoded into plasmid, linear or closed circular DNA, with cell-free extract, and a reaction buffer, the contents of which can be optimized (Sun et al., 2013a). For instance, the addition of maltodextrin (Wang and Zhang, 2009) and to a lesser degree maltose (Caschera and Noireaux, 2014) as an additional energy source can increase protein production, essentially prolonging the duration of *in vitro* reactions for up to 10 h.

**Figure 3:**
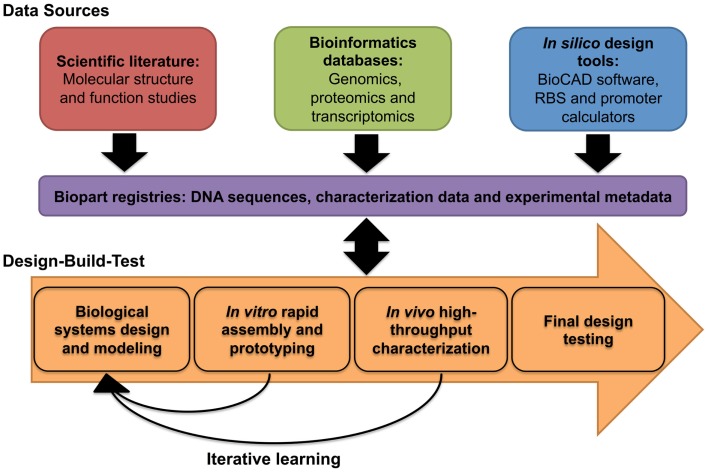
**Systematic design of biological systems**. The biological design cycle is one of several engineering principles that have been adopted in synthetic biology, and it describes the iterative process of designing a biological system through multiple rounds of design, build, and testing. To ensure that iterations of the design cycle are informative, the systematic capture, and integration of experimental and experiential data within a biological design workflow, such as the one shown here, is desirable.

Transcription–translation characterization systems provide characterization data within a timescale of hours (Chappell et al., 2013), and are therefore, amenable to a rapid prototyping workflow (Chappell et al., 2013; Sun et al., 2013b). For instance, Chappell et al. (2013) characterized a panel of Anderson constitutive promoters, using a commercially available TX–TL system, within a 5-h workflow. Interestingly, the *in vitro* characterization data of a set of Anderson promoters correlated with their performance *in vivo* (Chappell et al., 2013). Likewise, in the same study, a panel of LasR responsive, AHL-inducible promoters, also behaved similarly *in vitro* and *in vivo*, although meaningful comparisons could only be made where constructs were encoded into plasmid or closed circular DNA (Chappell et al., 2013). PCR-generated linear DNA templates did not produce sufficient transcription and translation of the reporter protein (Chappell et al., 2013). Based upon several reports, it is likely that linear DNA templates are unstable *in vitro* due to the presence of exonuclease activity in the cell-free extract (Sitaraman et al., 2004; Sun et al., 2013b). Expression of the phage lambda protein Gam, an inhibitor of RecBCD (ExoV), along with other modifications, can minimize linear DNA degradation, thus restoring protein expression to levels that are comparable to plasmid DNA constructs (Sitaraman et al., 2004; Sun et al., 2013b). Yet, in disagreement with several other studies (Chappell et al., 2013; Iyer et al., 2013; Lu and Ellington, 2014), Sun et al. (2013b) reported that *in vitro* characterization data were not comparable to *in vivo* data, though they did describe a methodology to calibrate between them. While the comparability between *in vitro* and *in vivo* characterization requires further investigation, several reports have demonstrated that cell-free TX–TL systems have proven utility in the rapid prototyping of logic-based genetic circuits (Karig et al., 2012; Shin and Noireaux, 2012; Iyer et al., 2013) or synthetic operons (Lu and Ellington, 2014). Within a systematic design context, *in vitro* characterization approaches have the potential to complement *in vivo* prototyping efforts by rapidly providing the characterization data required to rationally select a smaller number of designs for final testing (Figure 3).

## Conclusion

Synthetic biology is generally described as the “engineering of biology” yet since its inception, the field has faced the well-understood reality that biological systems are complex, stochastic, and difficult to predict, and are therefore, intrinsically difficult to engineer. In order to address these fundamental challenges, synthetic biology must use and explore the existing large body of knowledge of biological systems at different scales from molecular to cellular to organismal. By establishing a systematic design framework in which existing biological knowledge can be adapted and utilized will ensure the rapid development of successful applications using synthetic biology. Furthermore, the accumulated measurements and acquired knowledge of many synthetic biology experiments will allow synthetic biologists to establish design rules that tackle biological complexity, such that robust biological systems can be designed, assembled, and prototyped as part of a biological design cycle. At each stage of the design cycle, an expanding repertoire of tools is being developed. In this review, we have highlighted several of these tools in terms of their applications and benefits to the synthetic biology community within the context of the synthetic biology design cycle namely, designing predictable biology (design), assembling DNA into bioparts, pathways, and genomes (build), and rapid prototyping (test).

Design encompasses the development of tools and methodologies that make it easier to forward-design predictable synthetic biological systems. While there are several areas that are critical to designing predictable biology including, chassis selection, biopart design, or engineering strategies, as well as, several accompanying *in silico* design tools, we would argue that measurement and characterization (metrology) of biological parts, devices, and systems is essential for the field of synthetic biology to fulfill its promise. It is only through improvements in our ability to measure and generate meaningful conclusions about the behavior of biological processes that the field can progress in terms of unlocking additional biological design rules. The RBS calculator is the current exemplar of this perspective, though further work is required to equip the synthetic biology toolbox with the tools to make it easier to engineer radically complex synthetic biological parts, devices, and systems.

Build encompasses DNA assembly and genome-engineering methods that enable synthetic systems to be assembled. The field has benefited immensely from the BioBrick assembly standard. BioBrick assembly, effectively making bioparts reusable (modular) at the physical DNA level, creates a standard that enables multiple research groups to use and share an expanding library of bioparts, without the need for bespoke cloning strategies. While limitations in the BioBrick assembly standard led to the emergence of powerful overlap-directed assembly methods, including Gibson, these methods also shifted away from several of the core principals of synthetic biology since these methods rely on bespoke cloning strategies. However, emerging DNA assembly methods including MODAL or Gibson with UNSes, aim to unify the advantages of overlap-directed assembly with the engineering principle of modularity. However, advances in DNA synthesis and resultant reduction of costs could radically transform the field, such that more time could be diverted away from DNA assembly toward the designing or testing of synthetic systems.

Test encompasses elements of biopart characterization, since even the testing of non-functional designs may provide insights into our understanding of the biological design rules. Liquid-handling robot high-throughput characterization platforms, along with plate readers are equipped to test prototypes of synthetic bioparts, devices, and systems. However, these systems benefit from the addition of flow cytometry and microfluidics, which bring single cell analysis to these platforms. Thus, individual cells could be analyzed and selected based upon preferred biological performance from a heterogeneous cell mix. Additionally, an array of emerging *in vitro* TX–TL cell-free characterization systems provide characterization data within a timescale of hours, and are therefore, amenable to a rapid prototyping workflow. Such systems are complementary to *in vivo* high-throughput approaches and may speed up iterations through the design cycle by reducing the number of final designs that need to be tested.

As the synthetic biology toolkit expands and more design rules are unlocked, the most successful forward-design strategies are likely to be those that encompass a diverse workflow that combines several interoperable tools at each stage of the design cycle.

## Conflict of Interest Statement

The authors declare that the research was conducted in the absence of any commercial or financial relationships that could be construed as a potential conflict of interest.
